# Proteasome inhibitors in medullary thyroid carcinoma: time to restart with clinical trials?

**DOI:** 10.3389/fendo.2023.1145926

**Published:** 2023-04-20

**Authors:** Giuseppe Fanciulli, Roberta Modica, Anna La Salvia, Erika Maria Grossrubatscher, Tullio Florio, Francesco Ferraù, Alessandro Veresani, Flaminia Russo, Annamaria Colao, Antongiulio Faggiano

**Affiliations:** ^1^ Neuroendocrine Tumor Unit, Department of Medicine, Surgery and Pharmacy, University of Sassari, Sassari, Italy; ^2^ Endocrine Unit, Azienda Ospedaliero-Universitaria (AOU) Sassari, Sassari, Italy; ^3^ Endocrinology, Diabetology and Andrology Unit, Department of Clinical Medicine and Surgery, Federico II University of Naples, Naples, Italy; ^4^ National Center for Drug Research and Evaluation, National Institute of Health (ISS), Rome, Italy; ^5^ Endocrine Unit, Azienda Socio Sanitaria Territoriale (ASST) Grande Ospedale Metropolitano Niguarda, Milan, Italy; ^6^ Department of Internal Medicine, University of Genoa, Genoa, Italy; ^7^ Scientific Institute for Research, Hospitalisation and Healthcare Ospedale Policlinico San Martino, Genoa, Italy; ^8^ Department of Human Pathology of Adulthood and Childhood “G. Barresi” (DETEV), University of Messina, Messina, Italy; ^9^ Endocrinology Unit, Department of Internal Medicine and Medical Specialties (DiMI), University of Genoa, Genoa, Italy; ^10^ Endocrinology Unit, Department of Clinical and Molecular Medicine, The European Neuroendocrine Tumor Society (ENETS) Center of Excellence, Sant’Andrea Hospital, Sapienza University of Rome, Rome, Italy; ^11^ UNESCO Chair, Education for Health and Sustainable Development, Federico II University, Naples, Italy

**Keywords:** medullary thyroid carcinoma, proteasome inhibitors, bortezomib, carfilzomib, ixazomib, MG132

## Abstract

**Introduction:**

Medullary thyroid cancer (MTC) is a rare thyroid tumour whose management in advanced stages is challenging, despite effective therapeutic options having expanded in recent years. Proteasome inhibitors (PrIn) have shown the ability to improve patient outcomes, including survival and quality of life, in several malignancies, due to their ability to impair cell proliferation and cause apoptosis through the inhibition of the proteasome activity. Consequently, these drugs could represent a useful tool, alone or in combination with other treatments, in MTC patients.

**Aim of the study:**

This review aims to summarize the available *in vitro* and *in vivo* data about the role of PrIn in MTC.

**Materials and methods:**

We performed an extensive search for relevant data sources, including full-published articles in international online databases (PubMed, Web of Science, Scopus), preliminary reports in selected international meeting abstract repositories, and short articles published as supplements of international meetings, by using the following terms: medullary thyroid carcinoma, proteasome inhibitors, bortezomib, carfilzomib, ixazomib, delanzomib, marizomib, oprozomib, and MG132. Additionally, we conducted with the same keywords, an in-depth search in registered clinical trials repositories.

**Results:**

Our search revealed *in vitro* studies in human and murine MTC cell lines, based on the use of PrIns, both alone and in combination with other anticancer drugs, and two pertinent clinical trials.

**Conclusion:**

We found a strong discrepancy between the evidence of PrIns effects in preclinical studies, and the scarcity or early interruption of clinical trials. We might speculate that difficulties in enrolling patients, as happens in other rare diseases, may have discouraged trials’ implementation in favor of drugs already approved for MTC. However, given the concrete improvement in the comprehension of the molecular basis of PrIn effects in MTC, new clinical trials with accurate inclusion criteria of enrollment might be warranted, in order to ascertain whether this treatment, alone or in combination with other drugs, could indeed represent an option to enhance the therapeutic response, and to ultimately improve patients’ outcome and survival.

## 1 Introduction

Medullary thyroid cancer (MTC) is a neuroendocrine neoplasm originated by parafollicular cells in the thyroid gland, representing about 3-5% of thyroid cancers (1). MTC incidence is increasing up to 0.21 per 100,000 subjects, and prognosis is variable: increasing age and advanced stage of presentation are associated with worse survival, and genetic status is a strong predictor of outcomes and response to treatment (2, 3). MTC may be either sporadic or hereditary, as a component of the type 2 multiple endocrine neoplasia (MEN2A and MEN2B), or the related syndrome familial MTC (FMTC) (4). MTC presenting with loco-regional metastasis are detected already at diagnosis in up to 50% of patients, with distant metastasis in approximately 10–15%, and recurrence occurs in about 50% of patients (5). In locally advanced MTC, the 10-years survival rate is up to 100%, lowering at 20% in advanced stages, and no significant increase in patient survival has been reported in recent years (4). According to NCCN clinical guidelines, total thyroidectomy and dissection of cervical lymph node compartment, whose extension might vary in view of calcitonin (CT) circulating levels, is the standard treatment for sporadic or hereditary MTC (4).

Treatment of advanced and progressive MTC may require medical therapies, though the optimal treatment, dose and timing are debated (1). The multikinase inhibitors (MKIs) vandetanib and cabozantinib are approved for the management of metastatic progressive or symptomatic MTC (6). Since the crucial role of Rearranged During Transfection (RET) mutations in an relevant percentage of MTC cases (4), and the off-target toxicities often given by MKIs, two selective RET inhibitors, selpercatinib and pralsetinib have been evaluated and approved for patients with advanced or metastatic RET-mutant MTC, either somatic or germinal, and are currently the first lines of treatment for metastatic or progressive tumours presenting RET mutations (7–9). The availability of targeted therapies represents a turning point in the MTC therapeutic scenario. Nevertheless, a significant impact on survival is still lacking. Furthermore, treatment toxicities and resistance may often cause treatment failure or withdrawal (6). Indeed, according to guidelines, stable or slowly progressive MTC are not suitable for these therapies (10).

Therefore, there is the clinical need to identify the best sequences and therapeutic strategies, integrating MKIs and RET inhibitors with other treatment options. Among these, chemotherapy with doxorubicin has demonstrated strong preclinical activity in cell lines and murine models of MTC (11, 12). However, in clinical studies doxorubicin has shown variable efficacy (13–15).

Finally, somatostatin analogues (16) and the MTKI sunitinib (17) have shown encouraging results.

In the last decades, the improvement of biotechnology and molecular biology techniques allowed the identification of several mechanisms involved in cancer onset, development, and progression, radically changing the management of these conditions. Among these molecular targets, the cell proteasome has been identified as a potentially relevant one. The proteasome is a multimeric protein complex responsible for the intracellular degradation of damaged, misfolded or no longer needed proteins, enabling the recycling process (18). Ubiquitination (an enzymatic process that involves the bonding of an ubiquitin protein to a substrate protein) targets these unwanted proteins to the proteasome which degrades them into small oligopeptides.

Proteasome structure consists of a sequence of four rings, each of them formed by seven different protein subunits, in which the two outer rings are named α rings while the inner ones are called β rings (19). The α subunits have a structural role, regulating the access of proteins into and out of the inner core. The protease activity of the complex is due to three of the seven β subunits that compose the two inner rings, namely β1, β2, and β5. The remaining four β subunits (β3, β4, β6, and β7) are devoid of proteolytic activity (20).

Ubiquitinated proteins are recognized by the regulatory subunits of the proteasome complex, the ubiquitin chains are removed and recycled, and the protein is unfolded and translocated into the inner rings of the proteasome complex where it is cleaved into peptide products by the active protease subunits (21). The catalytic activity of these subunits is exerted as threonine proteases, in which the threonine within the active site forms a covalent ester bond with the N-terminal portion of the substrate. Then this intermediate is hydrolysed in small peptides and amino acids and released, regenerating the active form of the protease. Active β subunits have a highly efficient proteolytic activity since each of them possess the activity of the most diffuse cellular proteases. In particular, β1 is endowed with a caspase-like, β2 with a trypsin-like, and β5 with a chymotrypsin-like activity (22).

Proteasome activity plays a fundamental role in normal cells, and a very important role in the proliferation of malignant cells, because the accumulation of mis-folded proteins, abundant in such cells due to the high proliferative rate, leads to cell death by apoptosis (23). For this reason, small-molecule proteasome inhibitors (PrIns) were investigated in preclinical studies, demonstrating an apoptotic effect in cancer cells and *in vivo* murine models of cancer (24, 25). PrIns exert their inhibitory action predominantly on the chymotrypsin-like β5 catalytic subunit. Proteasome inhibition-mediated cell death has been shown to result from elevated apoptosis via multiple pathways, including a reduced activation of nuclear factor (NF)-κB activity due to the blockade of the degradation of the inhibitor IκB, the inhibition of cyclin turnover, affecting cell cycle progression, and stabilizing protein acting as tumour suppressors such as p53, c-Jun NH2-terminal kinase (JNK), and the Bcl-2 family of proteins (20, 23).

PrIns have been demonstrated to be highly effective for hematologic malignancies (**Table 1**) with an improvement of patients’ outcomes and a favourable safety profile (26).

**Table 1:** Current FDA/EMA approved PrIns.

Brand Name	Generic Name	Target	Indication(s)	FDA Approval (Year)	EMA Approval (Year)	Route of Administration
Velcade	bortezomib	slowly reversible inhibitor β5>β1>β2 subunits	first-line/ relapsed/refractory multiple myeloma and mantle cell lymphoma	2003	2004	intravenous or subcutaneous injections
Kyprolis	carfilzomib	irreversible inhibitor β5>β2/β1 subunits	relapsed/refractory multiple myeloma	2012	2015	intravenous injection
Ninlaro	ixazomib	reversible inhibitor β5>β1 subunits	relapsed/refractory multiple myeloma	2015	2016	oral

They have also been tested for solid tumours, as an attractive novel cancer chemotherapeutic modality (27). PrIns showed encouraging activity in preclinical models of many types of cancers as pancreatic, ovarian, breast or lung cancer, both as single agent and as well as if combined with other agents (above all with chemotherapy).

### 1.1 Aim of the study

In this narrative review, we aimed to collect and discuss the available data about the effects of PrIns in MTC, including studies in both preclinical and clinical settings.

## 2 Materials and methods

We performed an extensive search for relevant data sources, including full-published articles in international online databases (PubMed, Web of Science, Scopus), preliminary reports in selected international meeting abstract repositories (American Society of Clinical Oncology, European Neuroendocrine Tumor Society, European Society for Medical Oncology), and short articles published as supplements of scientific journals, by using the following terms: medullary thyroid carcinoma, proteasome inhibitors, bortezomib, carfilzomib, ixazomib, delanzomib, marizomib, oprozomib, and MG132. Additionally, we conducted an in-depth search for Registered Clinical Trials on Clinical Trials.gov registry, European Clinical Trials Database, and China Clinical Trials Register.

The search was last updated December 26, 2022.

## 3 Results

### 3.1 Studies *in vitro*

The effects of PrIns have been studied *in vitro* in several human and murine MTC cell lines. *In vitro* data are summarized in **Table 2**.

**Table 2:** Results of *in vitro* studies of PrIns (alone or in combination with other drugs) on MTC cell (results are listed in chronological order).

Drug(s) employed	MTC cell lines	Main results	Authors	Publication) (Year)
Bortezomib	Human: TT, DRO81–1, HRO85–1. Murine: 6-23 (clone 6)	Cytotoxic effect	Mitsiades et al.	2006
MG132	Human: TT, DRO81–1, HRO85–1	Cytotoxic effect	Mitsiades et al.	2006
Bortezomib + doxorubicin	Human: TT	Synergistic cytotoxic effect	Mitsiades et al.	2006
Bortezomib + IGF-1	Human: TT	Suppressive effect of IGF-1 on bortezomib-induced cytotoxic effect	Mitsiades et al.	2006
Bortezomib + MDM2-inhibitor nutlin-3	Human: TT	Synergistic cytotoxic effect	Ooi et al.	2009
Bortezomib + vandetanib	Human: TT	Non-antagonistic cytotoxic effect	Poruchynsky et al.	2011
MG132	Human: TT	Decrease in RET protein levels	Poruchynsky et al.	2011
Bortezomib + HDAC-inhibitor romidepsin	Human: TT	Synergistic effect on decrease of RET protein levels	Poruchynsky et al.	2011
Bortezomib + vandetanib	Human: TT	Value of enrichment of the proteasome pathway in overcoming resistance to vandetanib	Glassberg et al.	2020

1a) PrIn approved for human use (bortezomib), alone or in combination with other drugs.

The first study on the effects of PrIns was published by Mitsiades et al. in 2006 (28). They found that bortezomib induces cell death in human (TT, DRO81–1, HRO85–1), and murine (6-23, clone 6) MTC cells lines, at the concentrations well within those achieved clinically in bortezomib-treated patients.

The study reports two additional interesting experiments, both performed in TT cells, in which a) a strong synergistic effect of bortezomib with sub-lethal doses of the chemotherapeutic drug doxorubicin, and b) a suppressive effect of IGF-1 on bortezomib-induced cell death were reported.

The effects of the combination of bortezomib and vandetanib have been examined in two different studies

In 2011, Poruchynsky et al. (29) found in TT cells that vandetanib coupled with bortezomib had non-antagonistic effects in cytotoxicity assays. Besides, they showed that, contrarily to vandetanib, bortezomib was able to cause a marked reduction of RET proteins, and a reduction of RET mRNA concentrations.

In 2020, in an elegant experiment, Glassberg and co-authors (30) exposed TT cell lines to increasing concentrations of vandetanib, in order to generate vandetanib-resistant cell lines, and subsequently evaluated both vandetanib-sensitive and vandetanib-resistant lines. Both whole exome and RNA sequencing demonstrate increased expression of RET C634W in both cell lines, to a significantly greater extent in the drug-resistant line than in the sensitive line. In this experiment, they observed different expression of transcripts between the vandetanib-sensitive and vandetanib-resistant cell lines, including multidrug-resistance 1 (which confers drug resistance in other cancers) and autotaxin (promotes cell survival), and an enrichment of the proteasome pathway (as a potential candidate of growth suppression by vandetanib), which was validated via exposure of the cell lines to bortezomib, thus suggesting that PrIns can be a potential therapeutic strategy for overcoming resistance. p53 is inactivated in many malignancies through missense mutations or overexpression of the human homologue of MDM2 (Hdm2), an E3 ubiquitin ligase that ubiquitinates p53 promoting its proteasomal degradation. Nutlin-3 is a molecule with affinity for the p53-binding pocket of Hdm2 that can disrupt the p53-Hdm2 interaction and activate p53, inducing apoptosis. Nutlin-3 can exert a cytotoxic effect on 3 different lines of MTC (DRO81–1, HRO85–1) (31). Interestingly, experiments performed on TT cells show that the combination of Nutlin-3 and bortezomib elicits synergistic cytotoxic effects. Histone deacetylases (HDAC)-inhibitors induce cancer cell cycle arrest, differentiation, and cell death. Mechanisms of anticancer effects of HDAC-inhibitors are not uniform; they may be different and depend on the cancer type, HDAC-inhibitors, doses, etc. HDAC-inhibitors seem to be promising anti-cancer drugs particularly in the combination with other anticancer drugs (32). HDAC-inhibitors vorinostat, belinostat, romidepsin, and panobinostat and have been approved for hematological malignancies. Treatment of TT cells with vorinostat, belinostat, or romidepsin decreases RET proteins, and RET mRNA concentrations. Interestingly, in TT cells, combination of bortezomib and the MDM2-inhibitor romidepsin depresses RET proteins level at a greater extent than either drug separately (31), thus suggesting a different mechanism of action.

### 3.2 PrIn not approved for human use (MG132)

Very little data is available about MG132. The first use of the PrIn MG132 (carbobenzoxy-Leu-Leu-leucinal), a peptide aldehyde that effectively blocks the proteolytic activity of the 26S proteasome complex, dates back to 2006 (29). In this study, MG132 induced cell death in human MTC cell lines (TT, DRO81–1, HRO85–1). As for a possible mechanism of action, MG132 has been shown to cause a time and dose-dependent reduction in RET proteins in TT cells (30).

### 3.3 Clinical studies

Data on the use of PrIns in patients with MTC are limited to two studies, both using bortezomib, the first in combination with sunitinib, and the other with vandetanib (33, 34).

In the first study, published in 2013 (33), performed in 30 patients with refractory solid tumour malignancies treated with bortezomib and sunitinib, only 2 patients were affected by MTC. The primary objective of this phase 1 study was to establish the safety and determine the maximum tolerated doses of bortezomib and sunitinib in combination, dose-limiting toxicities and recommended doses of the drugs combination. Patients enrolled received bortezomib intravenously weekly and sunitinib orally daily for 4 weeks, followed by a 2-weeks rest. Initial doses were sunitinib 25 mg and bortezomib 1 mg/m2. Following dose selection was carried out according to the flexible Bayesian method of Escalation with Overdose Control (EWOC). The median number of cycles delivered was 3 (range 1-12). At the end of the study the recommended phase 2 dose of the combination was bortezomib 1,9 mg/m2 and sunitinib 37,5 mg. Common grade 3/4 toxicities were neutropenia, thrombocytopenia, hypertension, and diarrhoea. With regard to objective responses (ORs), 4 patients showed partial responses (PR) by RECIST criteria, and 6 patients stable disease (SD) > 6 months. Among the two patients with MTC, one showed PR, and one SD.

The Authors concluded that, given the results, “a phase 2 study of this combination in thyroid cancer patients is planned”. No further details about the type of thyroid cancer eligible for the phase 2 study (differentiated thyroid carcinoma, MTC, or both) are given. To date, no Phase 2 study is present in the clinical trials registries.

In the second study, published in 2019 (34), 22 patients with metastatic or advanced solid tumours (17 with MTC) were treated with vandetanib in combination with bortezomib. This phase I study was designed to evaluate the safety and tolerability of combined daily oral vandetanib and intravenous bortezomib on days 1, 4, 8, and 11 of an every 28-day cycle, to establish a recommended phase II dose of the drug combination. Patients enrolled received escalating doses of the 2 drugs for a median of 4 cycles with 13 patients escalating to 1,3 mg/m2 bortezomib/200 mg vandetanib and 10 to 1,3/mg/m2 bortezomib/300 mg vandetanib. The recommended phase II dose established at the end of the study was bortezomib 1,3 mg/m2 administered intravenously on days 1, 4, 8, 11, with oral vandetanib at a daily dose of 300 mg. Overall, the combination of the two drugs was administered safely with no G4/5 toxicities; the more frequent G3 toxicities observed were hypertension (24%), fatigue (19%), thrombocytopenia (10%), diarrhoea (10%), and arthralgia (10%). G3 grade prolonged QT interval was observed only in one patient. There was one dose-limiting toxicity, G3 thrombocytopenia. Among the patients with MTC, the Authors reported PR in 4/17 patients (23.5%). The decrease in CT appeared to correlate with RECIST response, but the correlation was limited (R2 = 0.54). No patient with SD duration of <6 months or progressive disease (PD) demonstrated a decrease in CT. The Authors concluded that the combination of the two drugs seem no better than those achievable with vandetanib alone, and decided to discontinue the planned phase II study, after having enrolled only one MTC patient.

## 4 Discussion

The preclinical evidence reported in this review are of some interest and might open new scenarios in the MTC treatment. The bortezomib-induced enhancement of doxorubicin effect may represent a potential therapeutic option in selected patients, and the observation that IGF-1 may exert a suppressive effect on bortezomib-induced cytotoxic effect might pave the way to studies based on the combination of bortezomib with somatostatin analogues. Besides, the use of bortezomib in MTC may be straightened by the results obtained with drugs not evaluated in patients with MTC, namely MDM2-inhibitors and HDAC-inhibitors. Finally, MG132 may represent in the future a new tool in this complex setting.

However, there are presently no published studies or ongoing RCTs (phase 1 or 2) employing doxorubicin, somatostatin analogues, MDM2-inhibitors, HDAC-inhibitors (in combination with bortezomib or other PrIns), or MG132.

As for the combination bortezomib/sunitinib and bortezomib/vandetanib, only two clinical trials are available, and they did not even reach Phase 2. The phase 1 study based on the combination of bortezomib and sunitinib (33), 2/2 patients with MTC had OR (1 PR and 1 SD), and the safety profile resulted similar to what was observed in other types of tumours. However, despite the plan to follow up with a phase 2 study, we cannot find any published study nor RCTs in the next 10 years. The phase 1 study based on the combination of bortezomib plus vandetanib (34), first posted on Clinical Trials.gov registry in 2009 (study registration number: NCT00923247), the decision to not pursued with the already planned Phase 2 study was taken by the Authors, as stated in the article, since they “felt this (study) might not be better than single-agent vandetanib with single-agent vandetanib with some added toxicity”. However, a detailed analysis of the figure reported in the study suggests a more favourable outcome, according to RECIST criteria: 18 patients, 5 of them with PR (27.8%), 9 showing SD (50.0%), and only with 4 PD (22.2%), even though SD may be little significant in a slow progressive disease within a short duration trial. This data, however, appears impressive when considering the characteristics of the MTC population: all patients with MTC had metastatic disease at the time of enrolment, 7 had prior radiation therapy, 6 systemic therapy, and 1 craniotomy for metastatic disease. As for the relatively low rate of adverse events, compared to those reported in literature (35) it is probably attributable to the presence of trial arms employing low doses of vandetanib, and to the short trial duration.

A (possible) placement of the combination bortezomib/vandetanib should take into account the recent European Medicine Agency indication (December 2022), according to which vandetanib should not be administered to patients in whom RET mutation status is not known or is negative (Caprelsa^®^ (vandetanib): Restriction of indication (aifa.gov.it), This restriction was based on data from the randomized study D4500C00058, and the observational study OBS14778, showing insufficient activity of vandetanib in patients with no identified RET mutations. Thus, in patients with negative or not known RET status, the combination therapy bortezomib/vandetanib might impact on the duration of tumour response, by decreasing the rate of escape observed in MTC patients initially responding to vandetanib. A further help in the decisional process of bortezomib placement in MTC therapy may be represented by the use of next-generation sequencing (NGS) gene panels, now accessible to clinicians (36), that will foreseeably reduce the number of non-mutated advanced MTC eligible for treatment with less selective treatments like PrIns. The strong discrepancy between evidence in preclinical studies and scarcity or early interruption of clinical trials may have several explanations, such as the recent availability of highly selective RET inhibitors, and the introduction of NGS in the selection of targeted drugs. However, we cannot exclude that the preclinical studies subsequent those that drove the RCT NCT00923247 could have been underestimated. If so, we believe that scientists should reconsider this class of drugs, to set off new strategies in this extremely challenging setting.

## 5 Conclusions

Available *in vitro* data support the possible efficacy of PrIns in MTC, while data from clinical trials are very limited. Only the inclusion of PrIns in clinical trials, with accurate inclusion criteria of enrolment, may ascertain whether this treatment, alone or in combination with other drugs, could indeed represent an option to enhance the therapeutic response, and to ultimately improve patients’ outcome and survival.

## Author contributions

GF, RM, AS, EG, TF, FF, AV, and FR were responsible for the design, the methodology, the draft preparation, the reviewing and editing. AC and AF were responsible for the supervision. All authors contributed to the article and approved the submitted version
